# Supplementary material for: Identification of photoperiod-induced specific miRNAs in the adrenal glands of Sunite sheep (Ovis aries)
Source: Front Vet Sci. 2022 Jul 22;9:888207. doi: 10.3389/fvets.2022.888207 (PMC9354845; doi:10.3389/fvets.2022.888207)
Supplement: Supplementary file 1 [file Data_Sheet_1.ZIP › Supplementary file/miRDeep2 file.zip!/miRDeep2 file/Novel_Structure/SPLP42A1/Novel_658.pdf]

Provisional ID	:	NC_019484.2_32512
Score total	:	1.9
Score for star read(s)	:	-1.3
Score for read counts	:	0
Score for mfe	:	1.6
Score for randfold	:	1.6
Score for cons. seed	:	
Total read count	:	743
Mature read count	:	743
Loop read count	:	0
Star read count	:	0

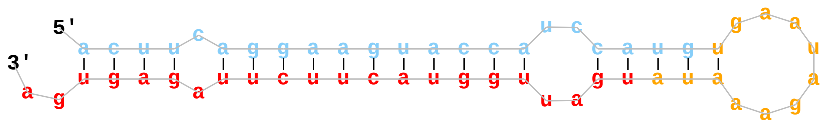

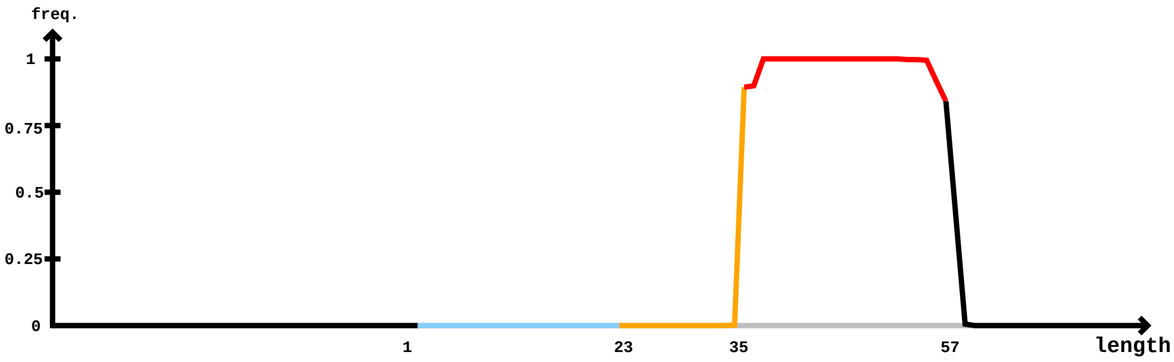

Star Mature

```
5'- ggucaccacauuucacuaugccgcgugcaguauugcacuucaggaaguaccauccaugugaauagaaauaugauugguacuucuuagagugaaguaaugcauagcaaauaca   -3'    exp
    (((................)))(((((((.((((.(((((.(((((((((((..(((((........)))))..))))))))))).))))).)))).))))).)).......     reads     mm     sample
    .....................................................................augauugguacuucuuagagu......................     1     0     seq
    ......................................................................ugauugguacuucuuag.........................     2     0     seq
    ......................................................................ugauugguacuucuuagag.......................     2     0     seq
    ......................................................................ugauugguacuucuuagagu......................     57    0     seq
    ......................................................................ugauugguacuucuuagagug.....................     55    0     seq
    ......................................................................ugauugguacuucuuagaguga....................     498   0     seq
    ......................................................................ugauugguacuucuuagagugaa...................     49    0     seq
    .......................................................................gauugguacuucuuagaguga....................     3     0     seq
    .......................................................................gauugguacuucuuagagugaa...................     1     0     seq
    ........................................................................auugguacuucuuagagug.....................     1     0     seq
    ........................................................................auugguacuucuuagaguga....................     45    0     seq
    ........................................................................auugguacuucuuagagugaa...................     25    0     seq
    ........................................................................auugguacuucuuagagugaag..................     4     0     seq
```
